# Supplementary material for: Common tissue-specific expressions and regulatory factors of c-KIT isoforms with and without GNNK and GNSK sequences across five mammals
Source: PLoS One. 2026 Jan 20;21(1):e0332294. doi: 10.1371/journal.pone.0332294 (PMC12818652; doi:10.1371/journal.pone.0332294)
Supplement: S1 Fig — Refer to the positions of the splice donor site (SDS) and splice acceptor site (SAS) in aligned RNA-seq sequence reads to distinguish between the shorter exon and the longer exon (A). Genomic locus of SDS1, SDS2, and SAS of KIT on the reference genome for each species (B). (PDF) [file pone.0332294.s001.pdf]

(A)

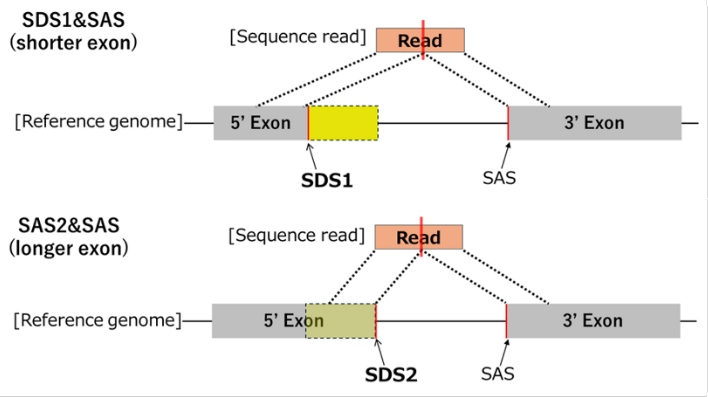

(B)

Gene	Species	SDS1	SDS2	SAS
KIT	*Homo sapiens*	chr4:54726039	chr4:54726051	chr4:54727217
	Mus musculus	chr5:75639147	chr5:75639159	chr5:75640492
	Canis lupus familiaris	NC_049234.1:47939125	NC_049234.1:47939137	NC_049234.1:47940332
	Felis catus	NC_058371.1:161318094	NC_058371.1:161318082	NC_058371.1:161316872
	Ovis aries	NC_056059.1:70933419	NC_056059.1:70933431	NC_056059.1:70934633

S1 Fig. Identification of isoforms. Refer to the positions of the splice donor site (SDS) and splice acceptor site (SAS) in aligned RNA-seq sequence reads to distinguish between the shorter exon and the longer exon (A). Genomic locus of SDS1, SDS2, and SAS of *KIT* on the reference genome for each species (B).
