# Supplementary material for: Common tissue-specific expressions and regulatory factors of c-KIT isoforms with and without GNNK and GNSK sequences across five mammals
Source: PLoS One. 2026 Jan 20;21(1):e0332294. doi: 10.1371/journal.pone.0332294 (PMC12818652; doi:10.1371/journal.pone.0332294)
Supplement: S2 Fig — Histograms of in–ex ratios across tissue types based on ENCODE tissue classification. (A) Mouse (Mus musculus), (B) dog (Canis lupus familiaris), (C) cat (Felis catus), and (D) sheep (Ovis aries). “n” indicates the number of samples for each histogram. The wavy line at x = 0 is where the expression of GN[N/S]K+ and GN[N/S]K − is equal. (PDF) [file pone.0332294.s002.pdf]

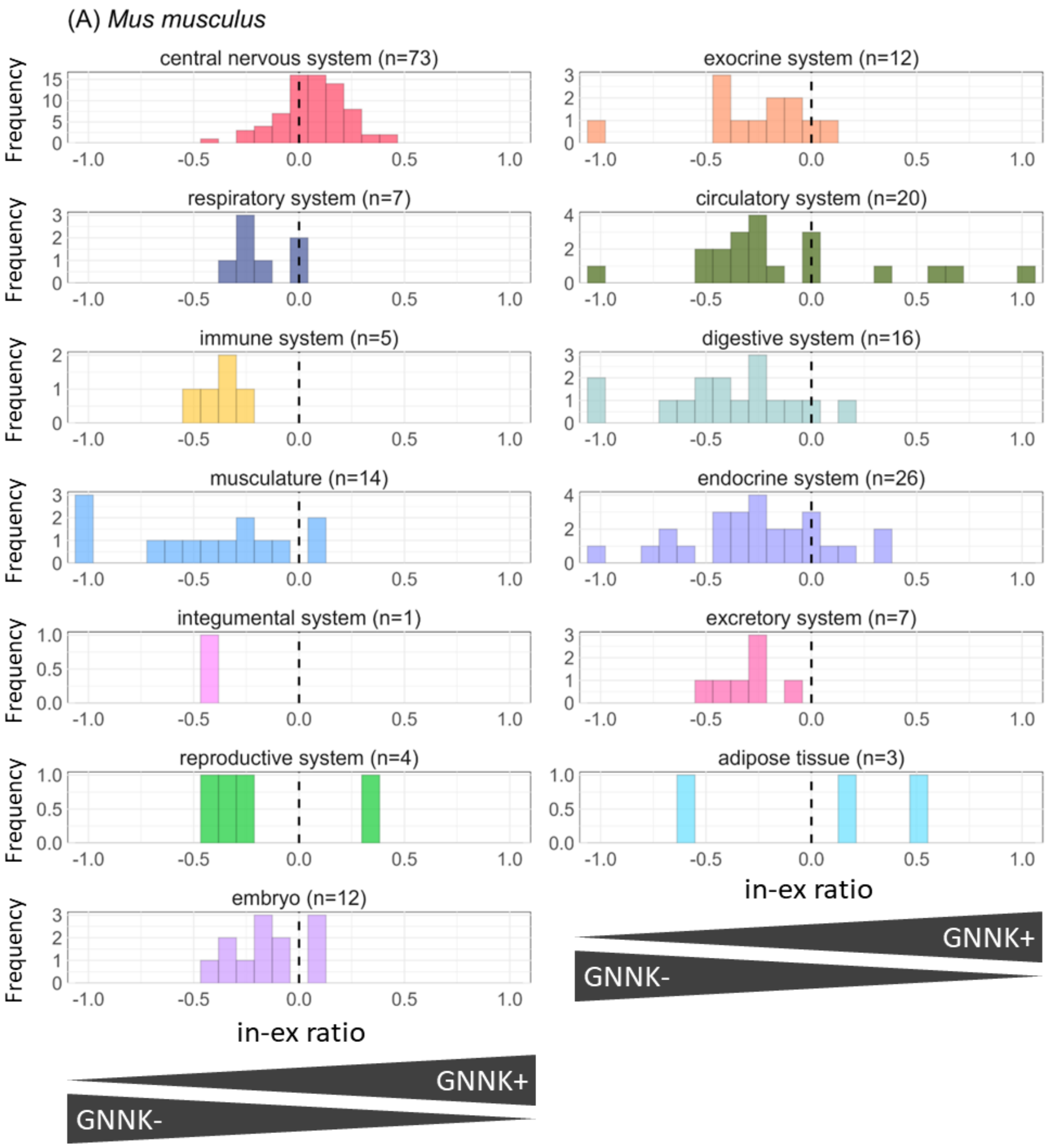

(A) Mus musculus
central nervous system (n=73)
exocrine system (n=12)
respiratory system (n=7)
circulatory system (n=20)
immune system (n=5)
digestive system (n=16)
musculature (n=14)
endocrine system (n=26)
integumental system (n=1)
excretory system (n=7)
reproductive system (n=4)
adipose tissue (n=3)
embryo (n=12)
Frequency
in-ex ratio
GNNK+
GNNK-

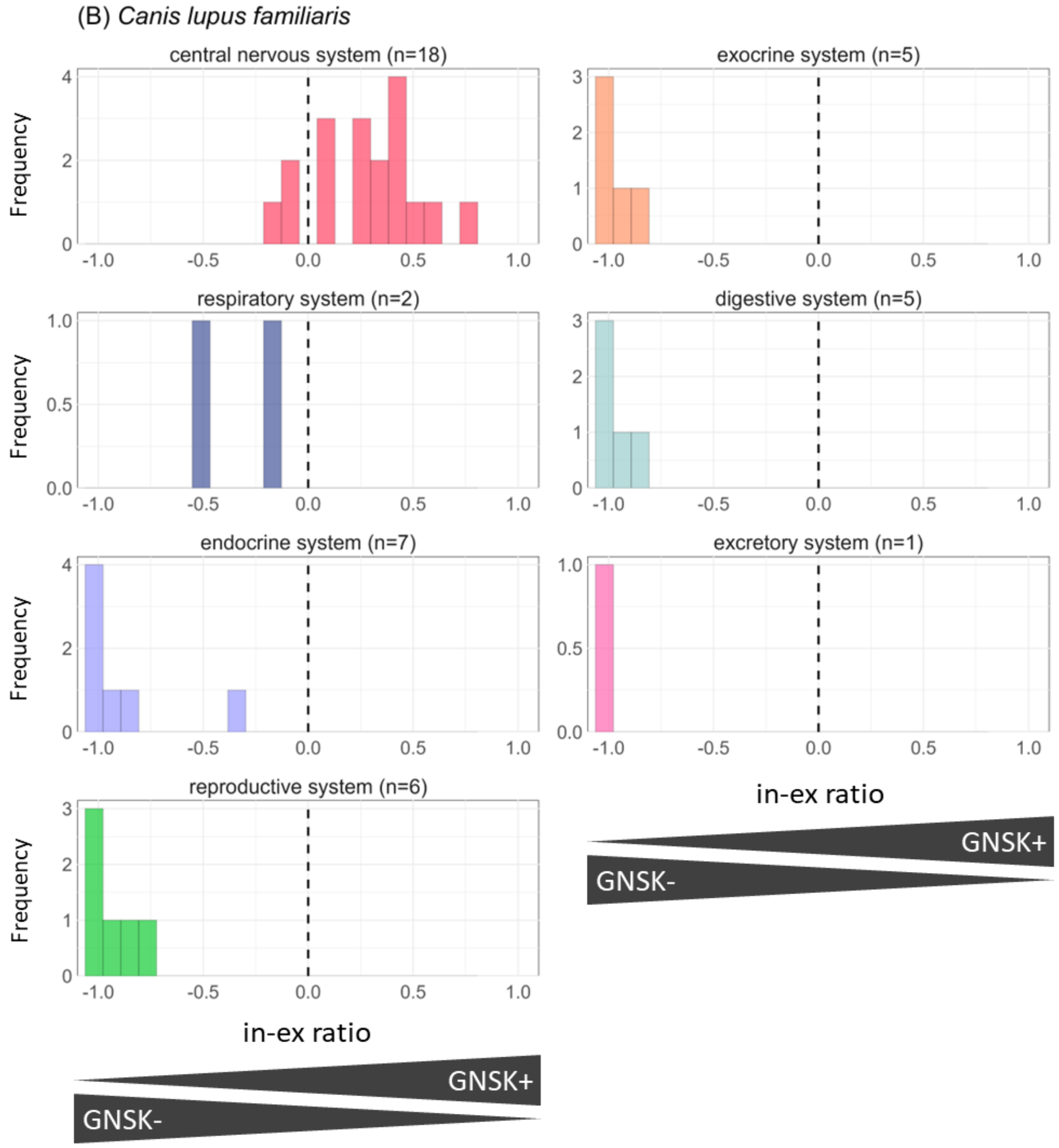
(B) Canis lupus familiaris
central nervous system (n=18)
exocrine system (n=5)
respiratory system (n=2)
digestive system (n=5)
endocrine system (n=7)
excretory system (n=1)
reproductive system (n=6)
Frequency
in-ex ratio
GNSK+
GNSK-

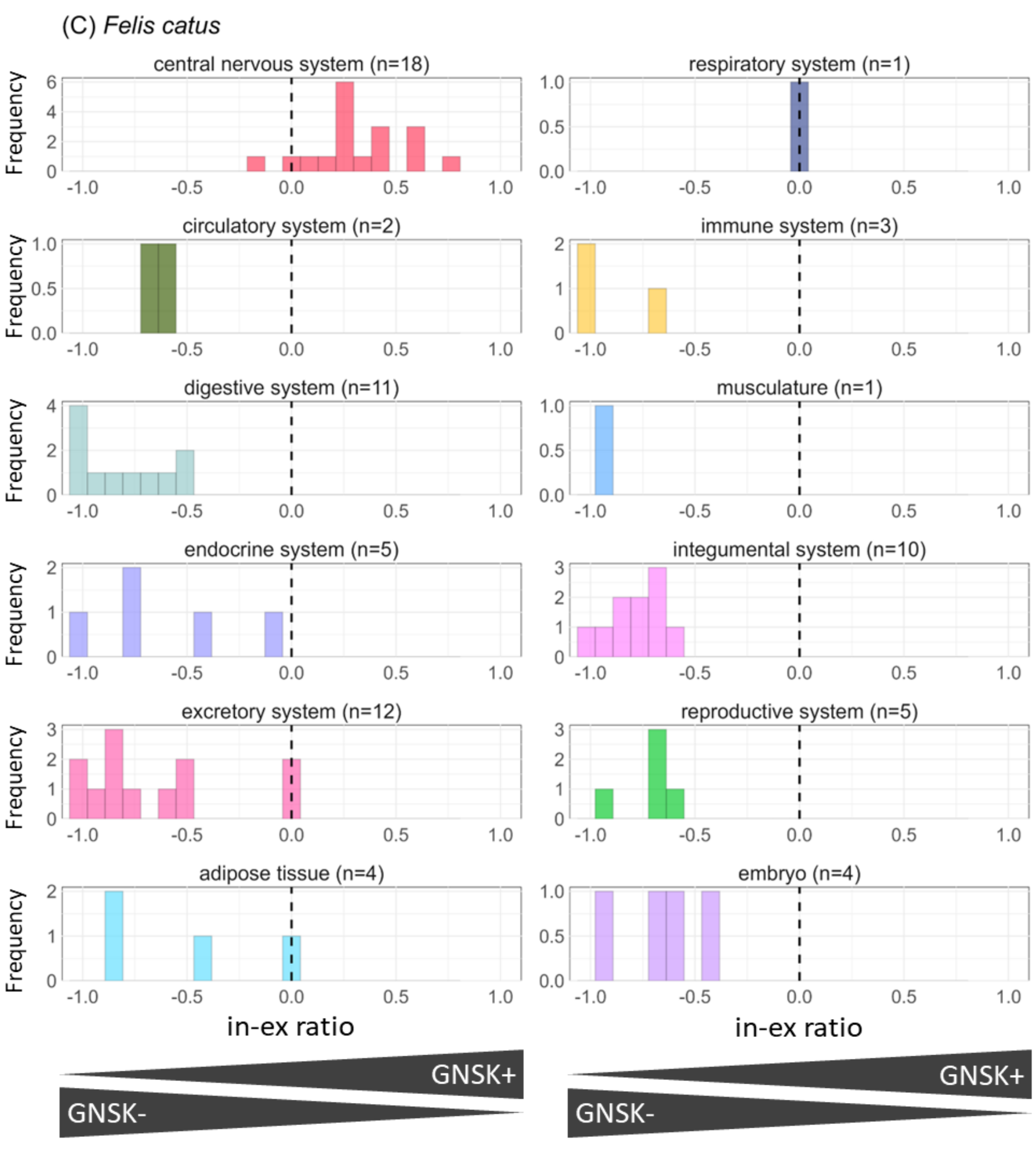

(C) Felis catus
central nervous system (n=18)
respiratory system (n=1)
circulatory system (n=2)
immune system (n=3)
digestive system (n=11)
musculature (n=1)
endocrine system (n=5)
integumental system (n=10)
excretory system (n=12)
reproductive system (n=5)
adipose tissue (n=4)
embryo (n=4)
Frequency
in-ex ratio
GNSK+
GNSK-

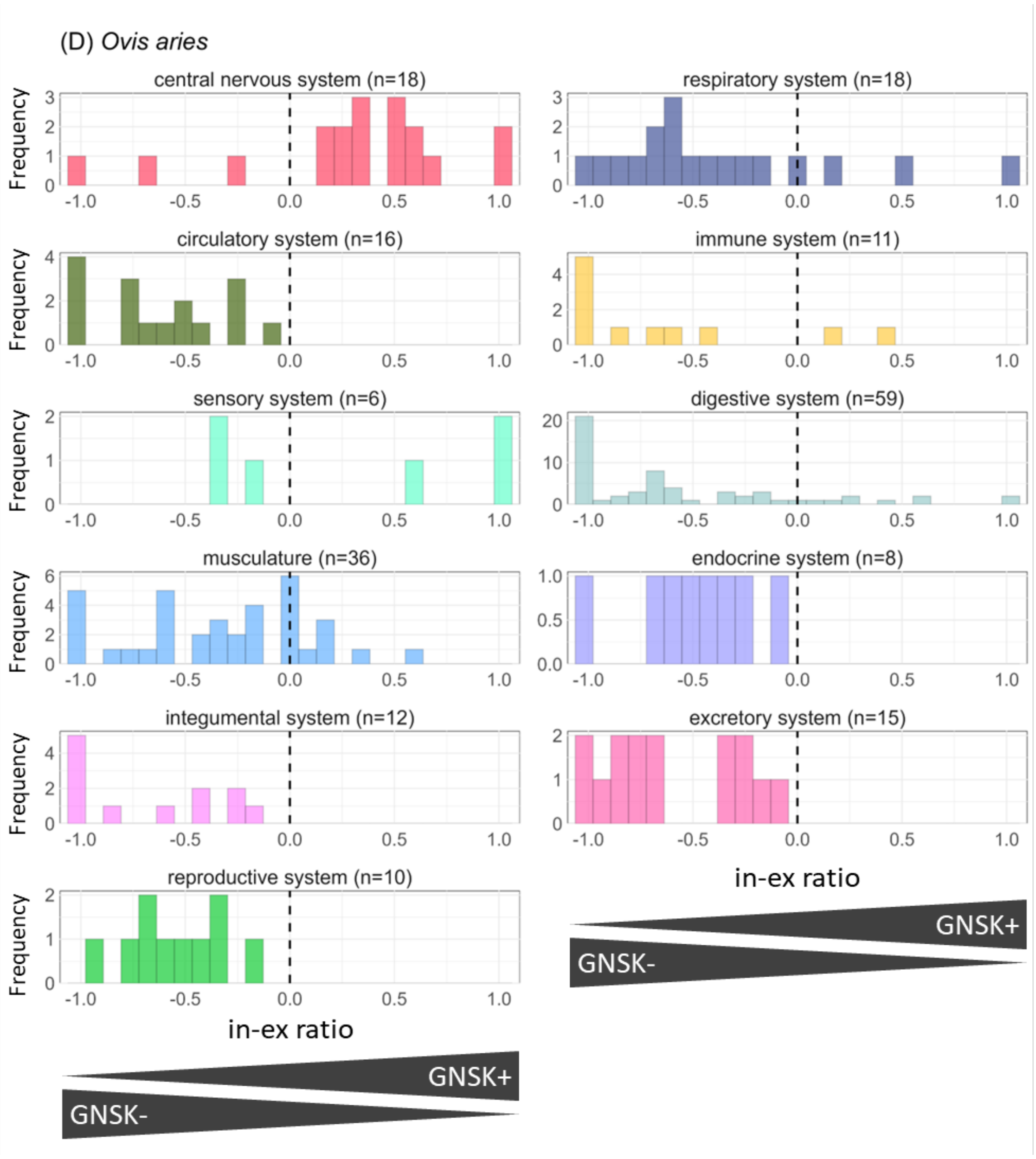

S2 Fig. Histograms of the in-ex ratio. Histograms of in-ex ratios across tissue types based on ENCODE tissue classification. (A) mouse (*Mus musculus*), (B) dog (*Canis lupus familiaris*), (C) cat (*Felis catus*), and (D) sheep (*Ovis aries*). The x-axis represents the in-ex ratio values, and the y-axis represents the frequency. "n" indicates the number of samples for each histogram. Draw a wavy line at x = 0, where the expression of GN[N/S]K+ and GN[N/S]K- is equal.
