# Supplementary material for: Common tissue-specific expressions and regulatory factors of c-KIT isoforms with and without GNNK and GNSK sequences across five mammals
Source: PLoS One. 2026 Jan 20;21(1):e0332294. doi: 10.1371/journal.pone.0332294 (PMC12818652; doi:10.1371/journal.pone.0332294)
Supplement: S3 Fig — The x-axis represents in–ex ratio values, and the y-axis represents the density calculated separately for CNS and other tissues. (A) Mouse, (B) dog, (C) cat, and (D) sheep. Red and blue bars indicate normalized numbers of CNS samples and other tissue samples exhibiting in–ex ratio values in each bin, respectively; the total areas of red and grey bars were each assigned 1. The wavy line at x = 0 is where the expression of GNNK+ and GNNK− is equal. (PDF) [file pone.0332294.s003.pdf]

(A) *Mus musculus*

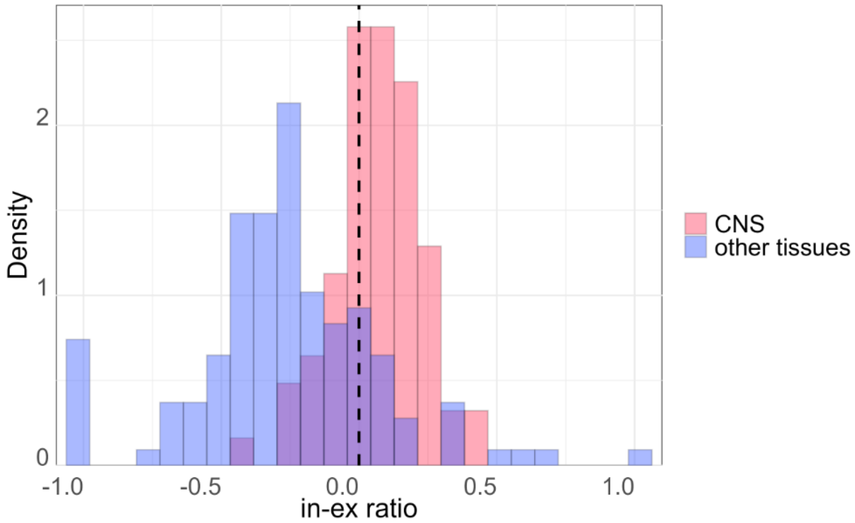

(B) *Canis lupus familiaris*

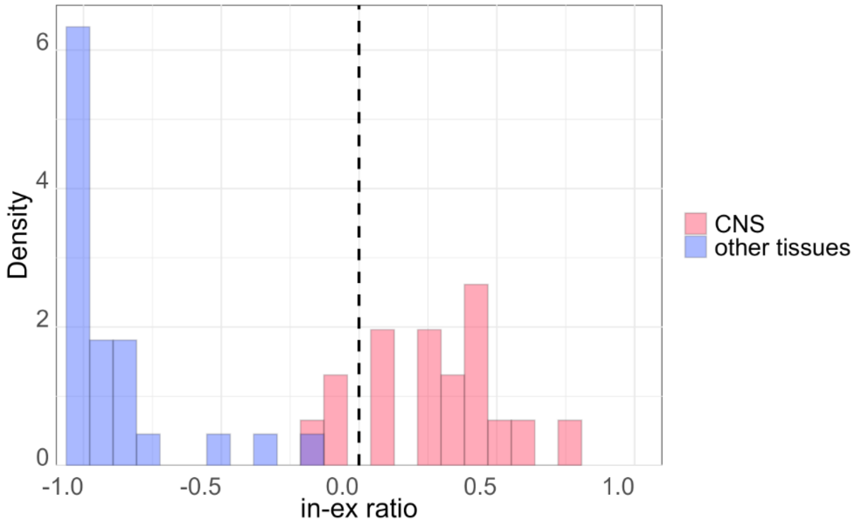

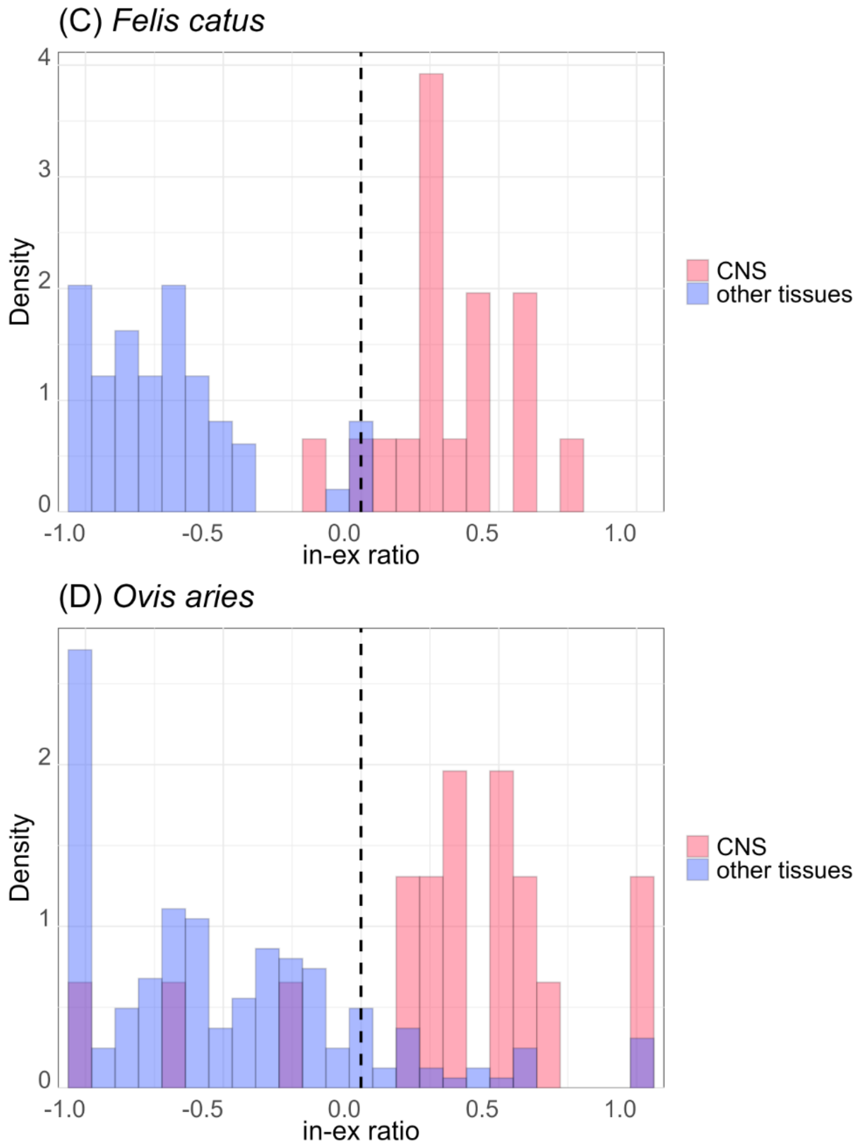

S3 Fig. Histogram of in-ex ratios colored by two categories: CNS and other tissues. The x-axis represents in-ex ratio values, and the y-axis represents the density calculated separately for CNS and other tissues. (A) mouse, (B) dog, (C) cat, and (D) sheep. Red and blue bars indicate normalized numbers of CNS samples and other tissue samples exhibiting in-ex ratio values in each bin, respectively; the total areas of red and grey bars were each assigned 1. Draw a wavy line at x = 0, where the expressions of GNNK+ and GNNK- are equal.
