# Supplementary material for: Common tissue-specific expressions and regulatory factors of c-KIT isoforms with and without GNNK and GNSK sequences across five mammals
Source: PLoS One. 2026 Jan 20;21(1):e0332294. doi: 10.1371/journal.pone.0332294 (PMC12818652; doi:10.1371/journal.pone.0332294)
Supplement: S4 Fig — Matrix of Wasserstein distance among histograms of in–ex ratios in different tissues was estimated for human (A), mouse (B), dog (C), cat (D), and sheep (E). The heatmap represents the Wasserstein distances, where each color bar range is independently scaled per species. A dendrogram was obtained by hierarchical clustering applied to a series of Wasserstein distances of each tissue. (PDF) [file pone.0332294.s004.pdf]

(A) *Homo sapiens*

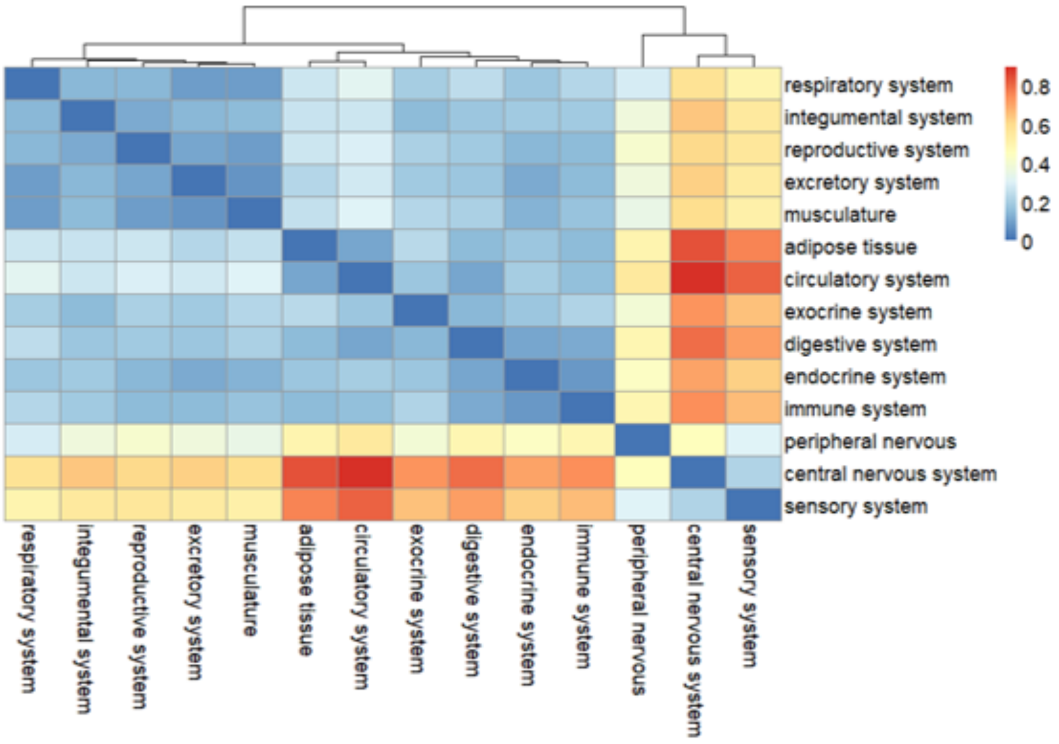


(B) *Mus musculus*

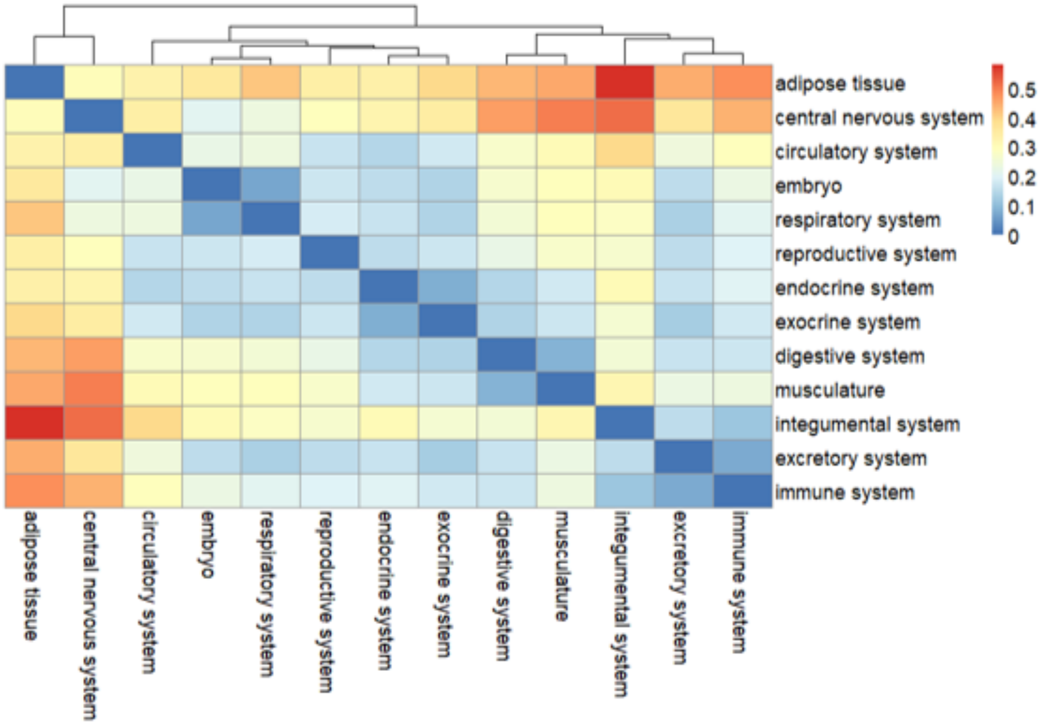

(C) *Canis lupus familiaris*

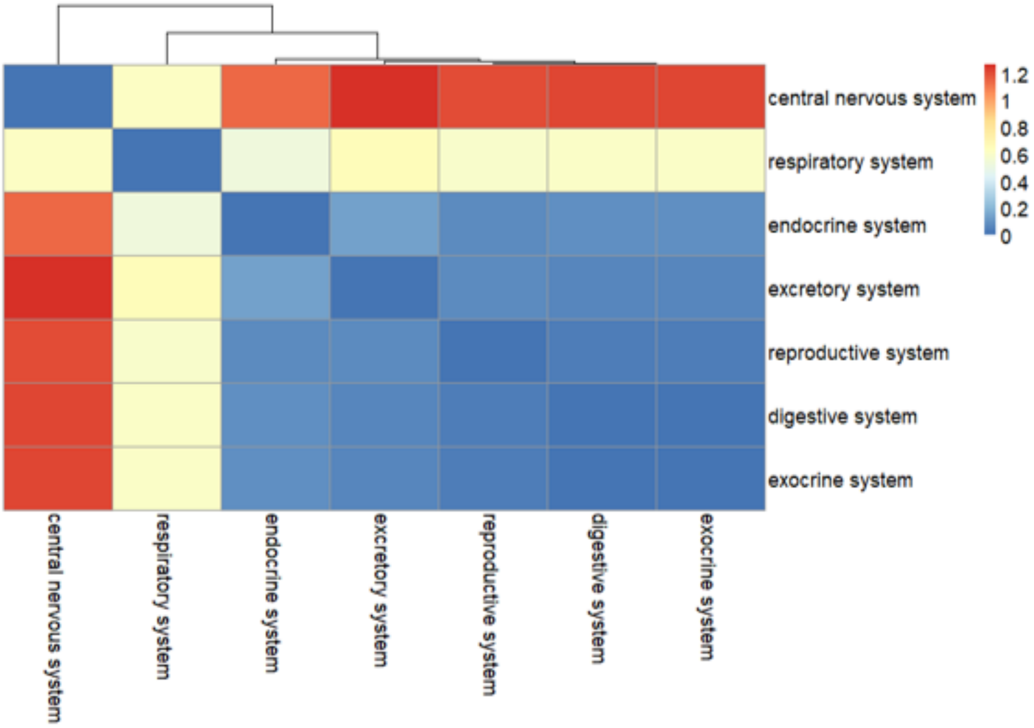


(D) *Felis catus*

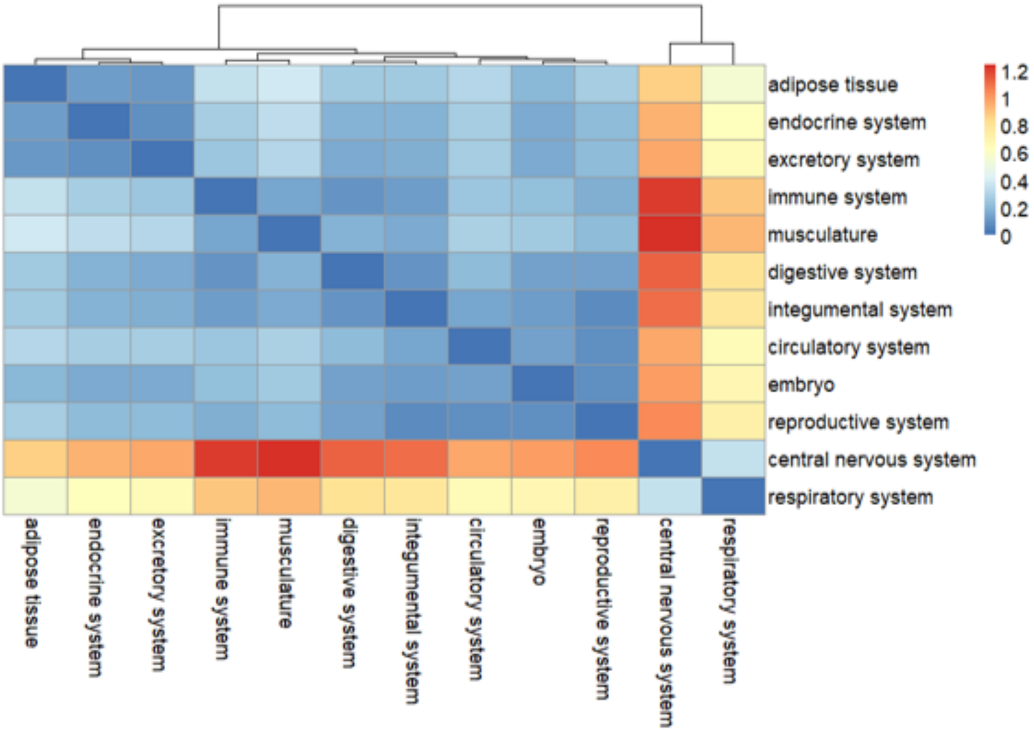

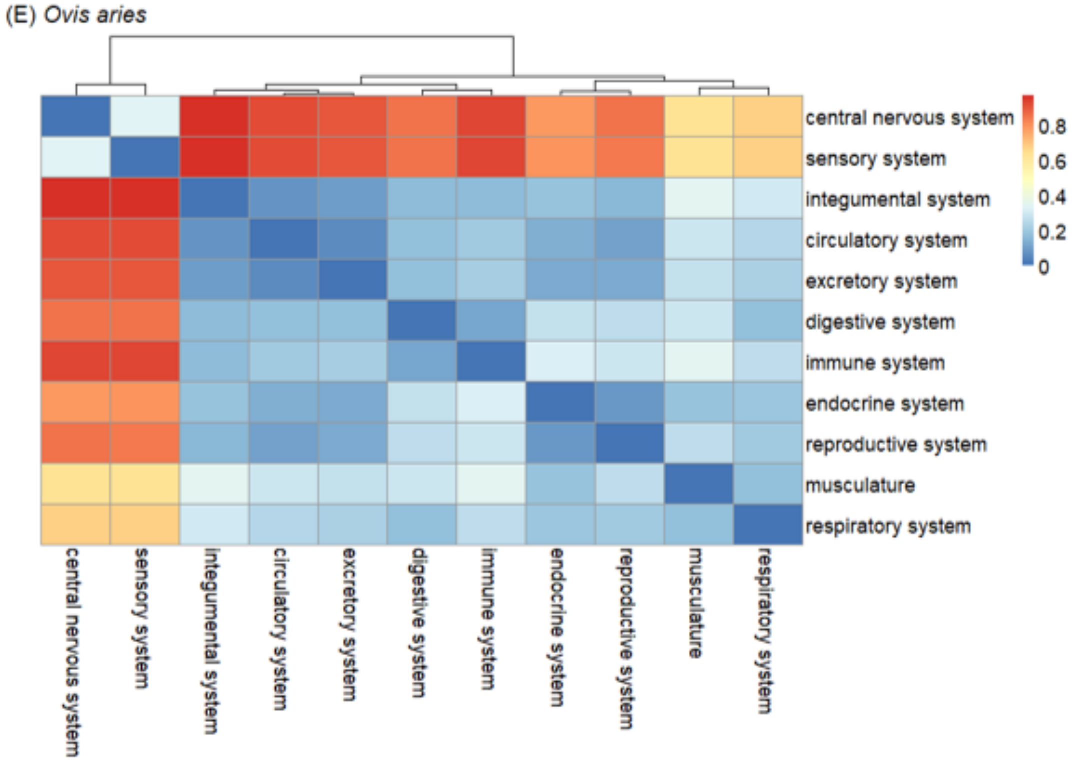


**S4 Fig. Matrix of Wasserstein distance.** Matrix of Wasserstein distance among histograms of in-ex ratios in different tissues was estimated for (A) human, (B) mouse, (C) dog, (D) cat, and (E) sheep. The heatmap represents the Wasserstein distances, where each color bar range is independently scaled per species. A dendrogram was obtained by hierarchical clustering applied to a series of Wasserstein distances of each tissue.
