# Supplementary material for: Common tissue-specific expressions and regulatory factors of c-KIT isoforms with and without GNNK and GNSK sequences across five mammals
Source: PLoS One. 2026 Jan 20;21(1):e0332294. doi: 10.1371/journal.pone.0332294 (PMC12818652; doi:10.1371/journal.pone.0332294)
Supplement: S5 Fig — Comparison of amino acid sequences of NOVA2 among human (NP_002507.1), mouse (NP_001025048.2), dog (XP_038278991.1), cat (XP_023100626.2), and sheep (XP_027834310.1). Sequence regions with black bars indicate KH domains. The red squares indicate positions with amino acid differences, and the numbers indicate their positions. Positions with asterisks represent identical amino acids among all five species. The amino acid sequences exhibited complete conservation among four mammals (human, dog, cat, and sheep), and mutations at seven amino acids were identified within the non-functional region of the mouse, situated between the second and third KH domains. (PDF) [file pone.0332294.s005.pdf]

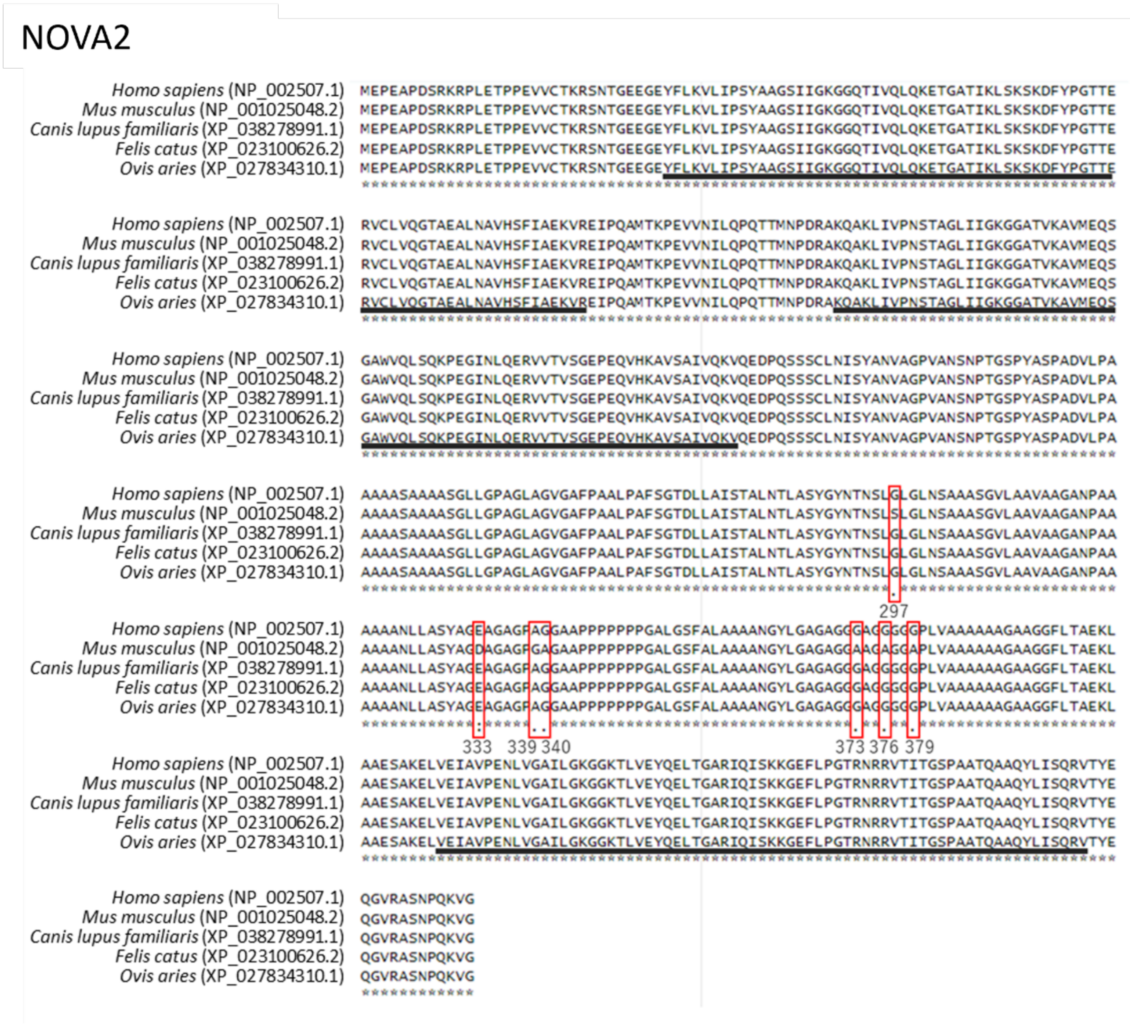

S5 Fig. Cross-species comparison of NOVA2 amino acid sequences. Comparison of amino acid sequences of NOVA2 among human (NP_002507.1), mouse (NP_001025048.2), dog (XP_038278991.1), cat (XP_023100626.2), and sheep (XP_027834310.1). Sequence regions with black bars indicate KH domains. The red squares indicate positions with amino acid differences, and the numbers indicate their positions. Positions with asterisks represent identical amino acids among all five species. The amino acid sequences exhibited complete conservation among four mammals (human, dog, cat, and sheep), and mutations at seven amino acids were identified within the non-functional region of the mouse, situated between the second and third KH domains.
