# Supplementary material for: Common tissue-specific expressions and regulatory factors of c-KIT isoforms with and without GNNK and GNSK sequences across five mammals
Source: PLoS One. 2026 Jan 20;21(1):e0332294. doi: 10.1371/journal.pone.0332294 (PMC12818652; doi:10.1371/journal.pone.0332294)
Supplement: S6 Fig — Sequence phylogenetic comparison of (A) the full-length KIT gene including introns and (B) the region encompassing the alternative exon, downstream intron and exon, using neighbor-joining tree method. Branch lengths above the branches represent evolutionary distances used to infer the phylogeny. (PDF) [file pone.0332294.s006.pdf]

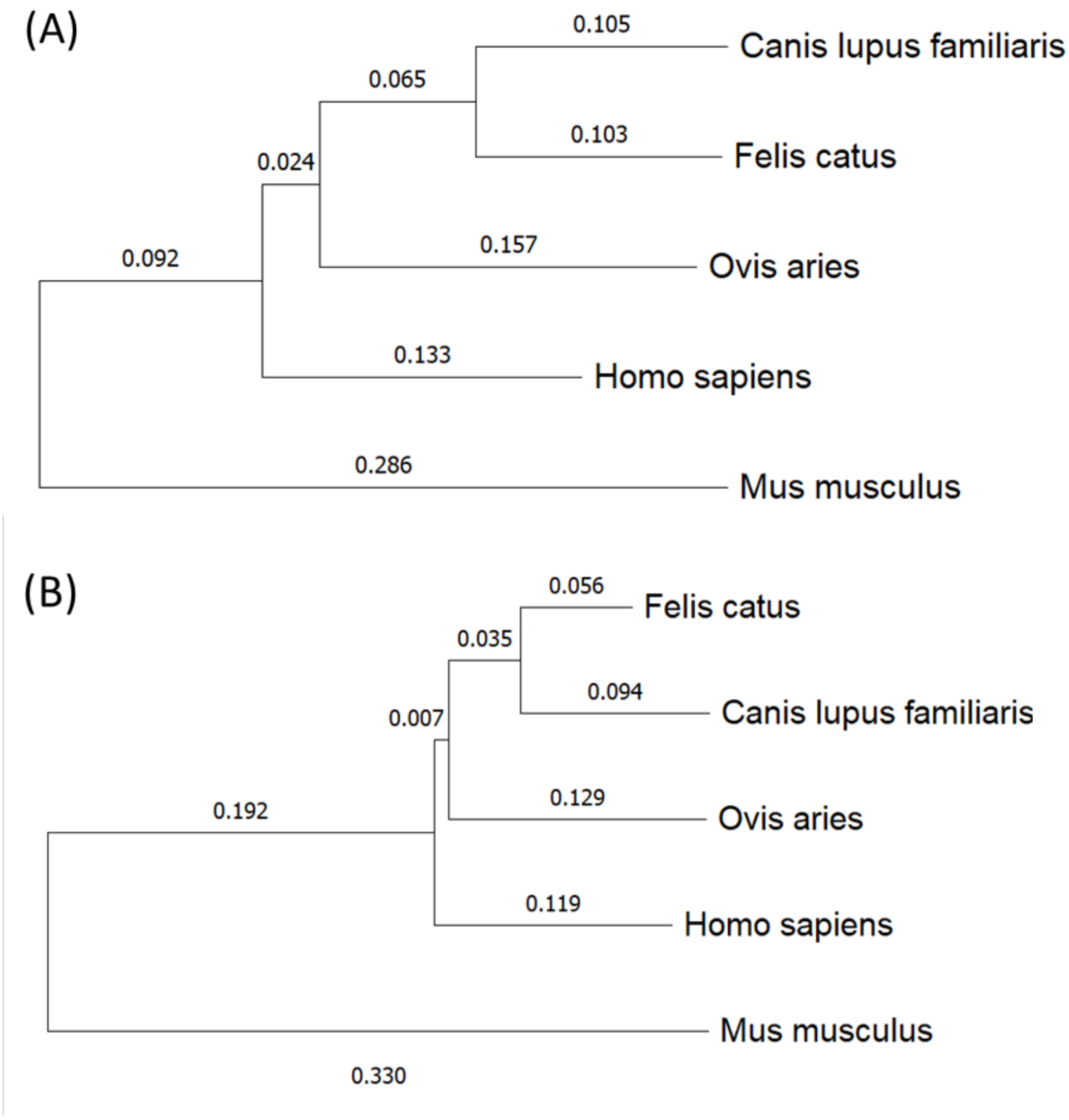

S6 Fig. Phylogenetic analysis of the *KIT* in five species. Sequence phylogenetic comparison of (A) the full-length *KIT* gene including introns and (B) the region encompassing the alternative exon, the downstream intron, and exon, using the Neighbor-joining tree method. The values indicate genetic distances.
